# Polygenic Risk for Insomnia in Adolescents of Diverse Ancestry

**DOI:** 10.3389/fgene.2021.654717

**Published:** 2021-05-10

**Authors:** Tengfei Ma, Honglei Chen, Qing Lu, Xiaoran Tong

**Affiliations:** ^1^Department of Epidemiology and Biostatistics, College of Human Medicine, Michigan State University, East Lansing, MI, United States; ^2^Department of Biostatistics, College of Public Health and Health Professionals and College of Medicine, University of Florida, Gainesville, FL, United States; ^3^Biostatistics and Computational Biology, National Institute of Environmental Health Sciences, Durham, NC, United States

**Keywords:** insomnia, adolescent, polygenic risk score, genetic, ancestry

## Abstract

**Background:** Insomnia is a common mental disorder, affecting nearly one fifth of the pre-adult population in the United States. The recent, largest genome-wide association study (GWAS) conducted on the United Kingdom Biobank cohort identified hundreds of significant single-nucleotide polymorphism (SNP), allowing the epidemiologists to quantify individual genetic predisposition in the subsequent studies *via* the polygenic risk scoring technique. The nucleotide polymorphisms and risk scoring, while being able to generalize to other adult populations of European origin, are not yet tested on pediatric and adolescent populations of diverse racial-ethnic backgrounds, and our study intends to fill these gaps.

**Materials and Methods:** We took the summary of the same United Kingdom Biobank study and conducted a polygenic risk score (PRS) analysis on a multi-ethnicity, pre-adult population provided by the Adolescent Brain Cognitive Development (ABCD) Study.

**Results:** The PRSs according to the significant nucleotide polymorphisms found in white British adults is a strong predictor of insomnia in children of similar European background but lacks power in non-European groups.

**Conclusions:** Through polygenic risk scoring, the knowledge of insomnia genetics summarized from a white adult study population is transferable to a younger age group, which aids the search of actionable targets of early insomnia prevention. Yet population stratification may prevent the easy generalization across ethnic lines; therefore, it is necessary to conduct group specific studies to aid people of non-European genetic background.

## Introduction

Insomnia is one of the most prevalent health complaints in medical practice and is marked predominantly by dissatisfaction with sleep quantity or quality (Substance Abuse and Mental Health Services Administration, 2016). The current criteria for the diagnosis of insomnia, according to the fifth edition of the *Diagnostic and Statistical Manual of Mental Disorders* (DSM), comprises difficulties falling asleep at bedtime and maintaining sleep and inability to return to sleep after early awakening in the morning, for at least 3 months (Roth, 2007; Substance Abuse and Mental Health Services Administration, 2016). This is different from the fourth edition of DSM, which has less stringent criteria for insomnia diagnosis, requiring the symptoms lasting only for 1 month (American Psychiatric Association, 2013). The estimated prevalence of insomnia in the United States is ranging from 15 to 24% (Pearson et al., 2006; Roth et al., 2011; Ford et al., 2015) depending on the diagnostic criteria.

Insomnia is also a common disorder among children and adolescents, with a prevalence of 10.7% (Johnson et al., 2006). Study found that sleep deprivation in children could result in abnormal neurobehavioral functioning and impaired brain development (Maski and Kothare, 2013), corroborated by the strong bidirectional association between neurodevelopmental disorders and insomnia (Dolsen et al., 2014). Compared with the general population, adolescents with insomnia have a higher chance of reporting comorbidities (Hiscock et al., 2015; Rigney et al., 2018). As childhood and adolescence are the most critical periods during the life course, when intellectual, physical, and psychological strengths grow rapidly and social perspective takes shape, identification of insomnia risk during this period would play an important role in the long-lasting disease-prevention efforts. Despite the high prevalence and projected significance, the genetic mechanism of insomnia in childhood adolescence remains poorly studied.

Recent genome-wide association studies (GWASs) of insomnia successfully identified 202 risk loci (Jansen et al., 2019). In this largest GWAS so far, the estimated single-nucleotide polymorphism (SNP) heritability was 7.0%, and the significant polygenic signal was observed (Jansen et al., 2019). To characterize the individual risk of insomnia due to genetics, this GWAS utilized the polygenic risk score (PRS) to summarize the impact of the entire genome. A PRS is the weighted sum of trait-associated alleles across many genetic loci of an individual, where the weights typically come from the effect sizes reported by a GWAS (Torkamani et al., 2018). Finally, the author reported up to 2.6% variation of the outcome (i.e., the onset of insomnia) explained by the PRS (Jansen et al., 2019). However, the generalizability of PRS could be affected by the heterogeneity between studies and target populations. A recent study found that the PRS derived from populations of European ancestry on which most GWAS conducted had poor performance in populations of African ancestry (Duncan et al., 2019). The poor performance could also be explained by the variation in linkage disequilibrium and allele frequency between different populations (Purcell et al., 2009; Duncan et al., 2019; Martin et al., 2019). Thus, we should consider population stratification when performing a PRS analysis.

Despite the stability of a person’s genetic material throughout his/her life span, and the fact that insomnia and related sleep disorders commonly appear in both adults and adolescents, the knowledge of the genetic mechanism learned from an adult population [i.e., the United Kingdom Biobank (UKB)] may not readily translate into the understanding of genetic impact on insomnia among the youths, knowing age is a strong surrogate of distinct biological processes (i.e., puberty vs. pre-puberty) and exterior exposures (i.e., hazardous workspace vs. school environment) that regulate the transcription of genetic materials associated with insomnia. This study is aimed to validate whether the PRS derived from adults of European ancestry could generalize to parent-reported insomnia severity scores in an adolescent cohort. We also examined the accuracy of this PRS in predicting other common sleeping disorders, including sleep breathing disorders, disorders of arousal, sleep–wake transition disorders, disorders of excessive somnolence, and sleep hyperhidrosis (Bruni et al., 1996). As we are aware of the adverse effect of population stratification on the prediction performance, we further evaluated the PRS among individuals of different ancestries.

## Materials and Methods

### Study Population

The baseline participants were adolescents aged 9–10 years from the Adolescent Brain Cognitive Development (ABCD) study. The study participants were recruited from 21 sites across the United States and will be followed up for 10 years. The goal of ABCD is to understand the effect of various factors such as stressful experiences and genetics on the brain, neurodevelopment, social, and emotional growth, and overall health (Hoffman et al., 2019). The study strives to represent a diverse range of socioeconomic and demographic profiles, reflecting the actual United States young generation as accurately as possible. Details of ABCD cohort design, recruitment, demographic makeup, and the assessments of physical and mental health are described elsewhere (Barch et al., 2018; Garavan et al., 2018). For our study of the PRS, a total of 11,875 children currently aged 9–11 years from ABCD were included.

### Outcome Assessment

Sleep Disturbance Scale for Children (SDSC) was administered to the parents to assess the sleeping disorders in their children (Bruni et al., 1996) with questions pertaining to child’s life in the past 6 months. SDSC contains 26 items rated on a Likert-type scale, e.g., “How many hours sleep does your child get on most nights?” (one indicates 9–11 h, two indicates 8–9 h, three indicates 7–8 h, four indicates 5–7 h, and five indicates < 5 h) and “The child has difficulty getting to sleep at night” (one indicates never; two indicates occasionally; three indicates sometimes; four indicates often; five indicates always). The sum of certain items consists the subscales that represent six common areas: (1) disorders of initiating and maintaining sleep (DIMS); (2) sleep breathing disorders (SBD); (3) disorders of arousal (DA); (4) sleep–wake transition disorders (SWTD); (5) disorders of excessive somnolence (DES); and (6) sleep hyperhidrosis (SHY). The items of DIMS in SDSC are close to the definition of insomnia diagnosis, so we consider DIMS as our primary outcome for insomnia.

### Genotyping

The sample preparation and genotyping are performed by Rutgers University Cell and DNA Repository (RUCDR). Of the 11,875 participants, 274 have no genotyping data. The genotyping of 11,601 participants was performed on Affymetrix NIDA SmokeScreen Array (Baurley et al., 2016), which contains 733,293 SNPs. Using PLINK, we retained 527,285 SNPs with a call rate greater than 99% across all participants.

We performed imputation on the Michigan Imputation server by selecting Hrc.R1.1.2016 of 40 million SNPs as the reference panel and Eagle v2.3 for phasing and using the multiethnic imputation process. After imputation, we retained SNPs with (1) an imputation INFO score higher than 0.9 (on a 0–1 scale), (2) a minor allele frequency (MAF) no less than 1%, (3) a Hardy–Weinberg equilibrium exact test *p*-value no less than 1 × 10^–6^, and (4) a call rate no less than 98%. We then excluded individuals with a missing rate higher than 2%. The quality control, again using PLINK, produced a working sample of 10,078 individuals with 3,919,675 SNPs.

### Polygenic Risk Score

Using PRSice-2 (Choi and O’Reilly, 2019), we clumped highly correlated SNPs with a 250-kb sliding window and an *R*^2^ threshold of 0.1. We then constructed the insomnia PRS based on the GWAS summary statistics provided by UKB, which were computed from 386,533 white British adults (Jansen et al., 2019). The PRS is a sum of reference allele counts of the ABCD SNPs, weighted by the test statistics (i.e., *z*-scores) from the UKB GWAS summary, which is the change in the log odds of developing insomnia per increment of reference allele. To reduce noise SNPs, we chose *a priori p*-value upper threshold of 0.01 as the inclusion criterion of ABCD SNPs. At this *p*-value threshold, the number of SNPs entered the calculation of PRS is 7,043 after the clumping procedure.

### Ancestry Assignment

Using principal component analysis (PCA) together with 2,502 high-quality reference genotypes representing five distinct ethnic groups from the 1,000 Genomes Project phase 3 (Auton et al., 2015), we estimated ancestry composition of five main ethnicities for our study samples. The PCA was performed by GCTA (v1.2.4) (Yang et al., 2011), which calculates the eigenvectors of the genetic relatedness matrix (GRM) derived from the imputed genotypes. We employed a simple linear mixed model to estimate genome-wide average proportions of European (EUR), African (AFR), East Asian (EAS), South Asian (SAS), and Native American (NAT) ancestries (Chen et al., 2020). We then assigned individuals to five broad ancestry groups using the estimated proportions and a significant threshold of 25%, which resulted in defining 6,371 European (≥25% EUR, < 25% AFR, < 25% EAS, < 25% SAS, and < 25% NAT); 1,209 African (≥25% AFR, < 25% EUR, < 25% EAS, < 25% SAS, and < 25% NAT); 182 East Asian (≥25% EAS, < 25% SAS, < 25% EUR, < 25% NAT, and < 25% AFR); 32 South Asian (≥25% SAS, < 25% EAS, < 25% EUR, < 25% NAT, and < 25% AFR); and 127 Native American (≥25% NAT, < 25% AFR, < 25% EAS, < 25% SAS, and < 25% EUR); the remaining 2,157 individuals were defined as admixed ancestry.

### Data Analysis

We use generalized linear mixed models (GLMMs) to investigate the association between the calculated insomnia PRS and insomnia severity score for each ancestry group separately. All the continuous outcome variables were standardized to *z*-score for better interpretation and comparison. Besides treating the PRS as fixed effect, all models included adolescent age, sex, and household income as covariates and included data collection sites and families as random effects. The missing value of the covariates was imputed by R package “mice” (Multivariate Imputation by Chained Equations) (Van Buuren and Groothuis-Oudshoorn, 2010). By using GLMM, we investigated the association between the PRS and other sleeping disorders such as SBD, DA, SWTD, DES, and SHY. The GLMMs were fitted using the R package lme4. To evaluate the impact of fixed effects including the PRS, we used marginal *R*^2^, which is the proportion of variance in the outcome (i.e., insomnia severity score) explained by the PRS alone (Nakagawa and Schielzeth, 2013). To account for multiple hypothesis testing, we calculated false discovery rate (FDR) separately for the primary analysis (18 tests in total) and the sensitivity analysis (12 tests in total), using the Benjamini–Hochberg procedure (Benjamini et al., 2001). A *q*−value (adjusted *p*-value) < 0.10 was considered statistically significant. We did not conduct analysis on Native American and Asian populations because of small sample sizes (127 and 214, respectively).

## Results

Description of sample characteristics, SDSC subscales, and PRS by three ancestry groups of ABCD participants are given in Table 1. *p*-value was calculated by ANOVA or Pearson’s chi-squared test. The mean insomnia PRS significantly differed by groups (*p* < 2.2 × 10^–16^). The participants of European ancestry have the lowest mean PRS for insomnia (mean = −0.159, SD = 0.60); the lowest SBD (mean = −0.110, SD = 0.84, *p* < 3 × 10^–16^), DES (mean = −0.028, SD = 0.92, *p* < 3 × 10^–6^), and SHY (mean = −0.026, SD = 0.95, *p* = 4 × 10^–3^); and the highest SWTD (mean = 0.018, SD = 0.97, *p* = 1 × 10^–3^). The participants of African ancestry have the highest DIMS (mean = 0.181, SD = 1.08, *p* < 2.0 × 10^–9^) and the lowest DA (mean = −0.021, SD = 1.29, *p* = 0.73).

**TABLE 1:** Participants characteristics, PRS, and SDSC subscales by ancestries.

	**European**	**African**	**Admixed**	*p*-value**
	Mean (SD)/*N* (%)*	**Mean (SD)/*N* (%)**	**Mean (SD)/*N* (%)**	
Age at assessment (month)	119.1 (7.5)	118.9 (7.2)	118.7 (7.5)	0.210
**Gender**				
Boy	3,394 (53%)	596 (49%)	1,095 (51%)	0.020
Girl	2,976 (47%)	610 (51%)	1,059 (49%)	
**Household income**				
Under $50,000	885 (15%)	698 (68%)	929 (49%)	<0.001
50,000 to $99,999	1,890 (31%)	219 (21%)	487 (26%)	
100,000 to $199,999	2,367 (39%)	92 (9%)	345 (18%)	
200,000 or above	899 (15%)	19 (2%)	125 (7%)	
PRS	−0.159 (0.6)	0.531 (1.9)	0.138 (1.1)	<0.001
**Sleep disorders**				
DIMS	−0.014 (0.99)	0.181 (1.08)	−0.018 (1.00)	<0.001
SBD	−0.110 (0.84)	0.396 (1.44)	0.095 (1.07)	<0.001
DA	0.002 (0.91)	−0.021 (1.29)	0.006 (1.08)	0.730
SWTD	0.018 (0.97)	−0.099 (1.13)	0.004 (1.00)	0.001
DES	−0.028 (0.92)	0.128 (1.30)	0.024 (1.05)	<0.001
SHY	−0.026 (0.95)	0.065 (1.17)	0.026 (1.01)	0.004

PRS, polygenic risk score; DIMS, disorders of initiating and maintaining sleep; SBD, sleep breathing disorders; DA, disorders of arousal; SWTD, sleep–wake transition disorders; DES, disorders of excessive somnolence; SHY, sleep hyperhidrosis. *Continuous data are presented as mean (SD), and categorical data are presented as *N* (%). **p-value for categorical data was calculated by Pearson’s chi-squared test, and *p*-value for continuous data was calculated by ANOVA.

Table 2 provides the results of the linear mixed models that evaluate the association between PRS and all six sleeping disorder scores by three ancestry groups. In the models for DIMS, we find a significant association among individuals of European ancestry. A one-unit increase in *z*-score for PRS of insomnia at GWAS SNP inclusion thresholds of *p*-value 0.01 is associated with a 0.066 *z*-score elevation of DIMS (*q*-value = 0.03). No association was found for other sleep disorder subscales and other two sub-populations of non-European ancestry.

**TABLE 2:** Summary of findings from linear mixed models of the insomnia polygenic risk score (PRS) and each sleep disorder subscale by different ancestries.

**Sub-scale**	**European**				**African**				**Mixed**			
	**Beta (95% CI)**	***R*^2^ (%)**	***p*-value**	*q*-value*	**Beta (95% CI)**	***R*^2^ (%)**	***p*-value**	***q*-value**	**Beta (95% CI)**	***R*^2^ (%)**	***p*-value**	***q*-value**
DIMS	0.066 (0.025, 0.106)	1.27	0.002	0.03	0.003 (−0.028, 0.035)	0.53	0.84	0.95	−0.021 (−0.060, 0.018)	0.32	0.30	0.64
SBD	0.001 (−0.034, 0.036)	1.51	0.96	0.96	−0.021 (−0.063, 0.022)	0.20	0.34	0.64	−0.027 (−0.069, 0.015)	1.17	0.20	0.61
DA	0.011 (−0.028, 0.049)	0.52	0.58	0.81	−0.008 (−0.046, 0.030)	0.47	0.68	0.88	0.003 (−0.040, 0.045)	0.61	0.90	0.95
SWTD	0.036 (−0.004, 0.077)	0.74	0.08	0.36	−0.015 (−0.048, 0.017)	0.78	0.36	0.64	−0.023 (−0.063, 1.581)	0.53	0.24	0.62
DES	0.037 (−0.001, 0.075)	1.73	0.05	0.32	0.015 (−0.024, 0.053)	0.81	0.46	0.69	−0.016 (−0.057, 0.025)	0.53	0.45	0.69
SHY	0.044 (0.004, 0.084)	1.82	0.03	0.26	−0.025 (−0.060, 0.009)	0.45	0.15	0.55	−0.003 (−0.042, 0.037)	2.18	0.89	0.95

Models are analyzed separately by ancestries. All models were adjusted by adolescent age, sex, and household income. Marginal *R*^2^ is used here to represent the proportion of variance explained by the fixed factors alone. *q-value is the FDR adjusted *p*-value.

**FIGURE 1:**
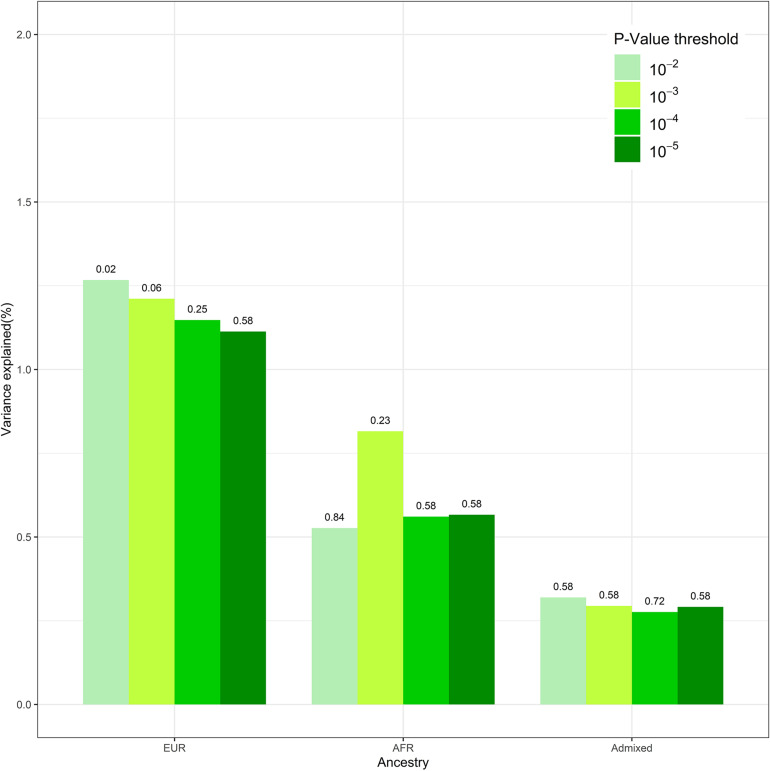
The polygenic risk score (PRS) performance in disorders of initiating and maintaining sleep (DIMS) by different ancestries. This figure compared the performance of the models at different genome-wide association study (GWAS) *p*-value thresholds. The numbers above the bar represent the *q*-value for PRS at each model.

We conducted sensitivity analysis on DIMS with PRS derived from different GWAS *p*-value thresholds. After the clumping procedure, the numbers of SNPs used to calculate PRS are 1,482, 359, and 155 under GWAS *p*-value of 1 × 10^–3^, 1 × 10^–4^, and 1 × 10^–5^, respectively. These findings were overall consistent (Figure 1). Insomnia PRS is significantly associated with DIMS in the European ancestry group when the *p*-value threshold was 0.01 or 0.001. The association became less significant with smaller *p*-value thresholds. The insomnia PRS explained the highest 1.3% variance at *p* = 0.01. No association between the PRS and DIMS was found in African and admixed ancestry groups across different *p*-value threshold.

## Discussion

We found the PRS derived from the GWAS report of white British adults a strong predictor of insomnia risk in white adolescents. The result is corroborated by previous epidemiologic evidence that childhood insomnia and related negative behavior patterns are significant precursors of adulthood sleeping disorder, hinting that the genetic causality sought out in an adult population should partially, if not entirely, generalize to the pre-adults. As a comparison, the original GWAS of UKB also repeated PRS analysis three times by randomly holding out 1,000 white British participants as testing datasets (Jansen et al., 2019); the result is a predictive power (*R*^2^ = 2.6%) higher than the PRS of the current study population of adolescents (*R*^2^ = 1.3%). The better performance of PRS in adults in terms of *R*^2^ could be explained by the different age population and insomnia measurements. In UKB, insomnia complaints were assessed by asking “Do you have trouble falling asleep at night or do you wake up in the middle of the night?” while in ABCD study, the DIMS subscale score is the sum of seven questions related to initiating and maintaining sleep. The sensitivity analysis gave the most predictive PRS under a GWAS *p*-value threshold of 0.01 (Figure 1); this may suggest that the genetic effect on insomnia is distributed across a wider range of low-effect variants across the genome.

The studies of twins estimated some moderate 30–60% heritability for insomnia, sleep, and related symptoms in both adults and children (Gehrman et al., 2011; Hublin et al., 2011; Lind and Gehrman, 2016), which suggests the prospect of identifying targets of prevention for healthy individuals at high risk or targets of intervention for people already experiencing insomnia. When a critical portion of heritability is captured by GWAS, as well as the PRS built upon the GWAS summary statistics, a carefully designed Mendelian randomization using PRS as a random “treatment” should be able to detect actionable, non-genetic items to either reduce the life-long risk of developing insomnia or mitigate the negative impact of onset cases.

On the other hand, we found a lack of power of the PRS to predict adolescent insomnia in non-white groups. An immediate suggestion would be the population stratification of genetic effects, attributed to heterogeneities in genotype patterns across racial/ethnic lines, or the interaction/competition between genetics and environment linked with group membership (i.e., social economics). For a wide range of disease and health outcomes, as reported, separately applying GWAS summary statistics of the White British in the UKB to European (EUR) and African American (AMR) in the 1,000 Genomes Project resulted in significant shift of PRS distributions (Duncan et al., 2019). Also, the PRS realized in the three sub-populations of ABCD are significantly different (Table 1, *p*-value < 0.001), further suggesting the existence of population stratification in terms of genotype heterogeneities across ancestries. Nonetheless, a GWAS conducted on the over-represented white population is not meant to dissect either type of population stratification effect. To improve the generalization of PRS on non-white populations, we require high-quality ethnic specific genetic association studies on insomnia, which in turn ask for an increased representation of diverse populations in genotyped cohorts with sample sizes on par with UKB.

With PRS, we cost-efficiently transferred the knowledge of genetic mechanisms gained from the high-quality adult cohort (i.e., UKB) to our target cohort of adolescents. However, even if the calculation of PRS was perfect, its performance in forecasting the risk of insomnia is limited by the percentage of heritability captured by the upstream GWAS. By the time of this study, the most comprehensive GWAS on insomnia only explained a fraction of the 30–60% heritability expected by twin family studies. We expect a better predictive power of the PRS along with the improvement in GWAS.

## Data Availability Statement

The data analyzed in this study is subject to the following licenses/restrictions: Registered researchers may access United Kingdom-Biobank and Adolescent Brain Cognitive Development (ABCD) subject genotype, diagnosis, and demographics. Requests to access these datasets should be directed to https://nda.nih.gov/abcd/request-access; https://www.ukbiobank.ac.uk/enable-your-research/apply-for-access.

## Author Contributions

TM designed the study. TM and XT performed the analysis and drafted the manuscript. QL applied for the access of ABCD dataset. QL and HC provided expertise on study design and analysis. All authors contributed to the article and approved the submitted version.

## Conflict of Interest

The authors declare that the research was conducted in the absence of any commercial or financial relationships that could be construed as a potential conflict of interest.
